# Comment on: “Analysis of Silicones Released from Household Items and Baby Articles by Direct Analysis in Real Time-Mass Spectrometry” by Jürgen H. Gross. *J. Am. Soc. Mass Spectrom.* 26, 511–521 (2015)

**DOI:** 10.1007/s13361-016-1334-z

**Published:** 2016-05-19

**Authors:** Karluss Thomas

**Affiliations:** Global Silicones Council, Washington, DC USA

In a recent paper, Gross [1] reported the release of silicone oligomers from articles of daily use by their exposure to a direct analysis in real time (DART) ion source and expressed concern for a substantial dose of silicones available for human intake. Although the results of the article clearly demonstrate that DART-MS may be used as a qualitative tool to identify silicone rubbers, there appear to be major errors introduced to the quantitation of silicone species by the calibration method employed. Additionally, the report considerably understates that the amount of polydimethylsiloxane (PDMS) observed after exposure of silicone materials directly to the DART source at 300 °C is substantially higher than what is released under normal use conditions.

## Determination of a Calibration Curve

The author cites four manuscripts that are excellent examples of quantitative DART analysis [2–5]. The experiments detailed in these papers are successful in providing quantitative analyses due to the details of the sampling geometry. Specifically, the cited experiments focus on liquids deposited onto the end of a capillary tube. Both the quantification standards and the samples slated for analysis are prepared in the same manner, providing a reproducible sample volume that has cross-sectional dimensions that are smaller than the dimensions of the DART metastable gas stream. As a result, in all four papers cited, either linear calibration curves (r^2^ ≥ 0.99) are presented or excellent sampling reproducibility is demonstrated (<4% coefficient of variance). In contrast, the author of this paper exposes real-world materials, which have variable geometries that are different than the quantification standards used to the DART gas stream. Thus, the size and shape of each sample is different, and presumably none of the samples has the same dimensions as the calibration standards. According to the text of the article and to Figure S3, the ionization source was mounted at a 45° angle relative to the detector and the sample was manually inserted into the beam. To build a calibration curve, “spots of several microliters were applied to a glass slide. The solvent was allowed to evaporate and the residual silicone oil spot was… [analyzed].” The author does not discuss the dimensions of the silicone oil spot that resulted from this deposition method. Nor does he explicitly discuss the dimensions of the DART gas stream. However, in the text of the article, he states, “The objects analyzed had a larger spatial expansion than such a spot and, thus, sample ions may have been generated and collected from an even larger surface, which in turn caused a higher amount of sample ions per run than could be obtained from a silicone oil spot. The sample spot size effect was also observed during the analysis of the SGE yellow silicone septum that was only 5 mm in diameter and was, thus, found to release only 20 μg of PDMS per run” [1, p. 517–18]. What we believe the author is saying is the following:
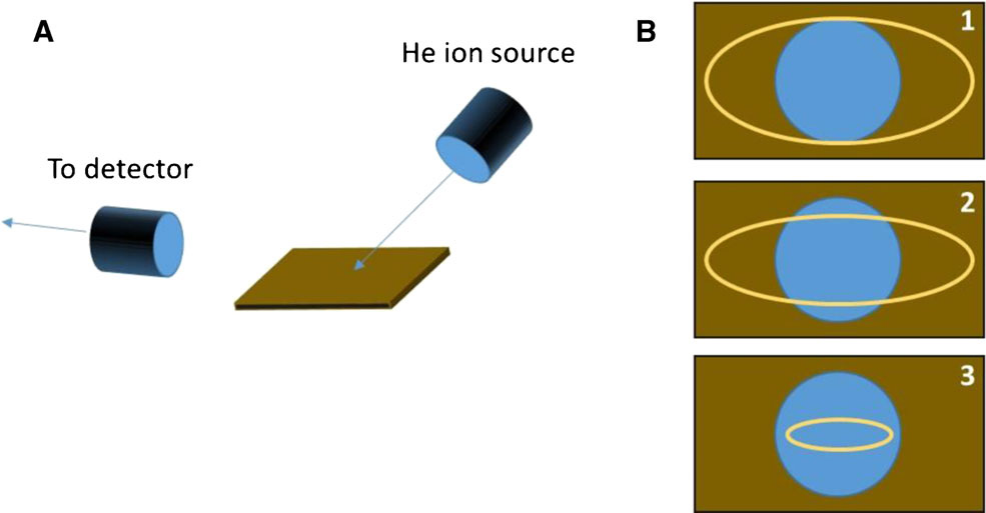


Figure A shows the source/sample/detector geometry of this experiment. The angle between the source and sample dictates that there will be an elongation of the beam as projected on the surface. Figure B shows an illustration of the shape of the DART gas stream (yellow oval) and the shape of the residual silicone oil spot (blue circle). Depending on the drop volume, gas stream dimensions, and relative height of the sample versus the source, three scenarios are possible: (1) The entire volume of the silicone oil will be sampled by the gas stream, and the gas stream will also sample additional portions of the glass. This is true if the gas stream is wider than the silicone drop. (2) The gas stream will sample some fraction of the silicone drop, along with additional portions of the glass. This is true if the gas stream is longer and narrower than the drop width. (3) The gas stream will sample only the silicone drop. This is the case if the gas stream length and width are both smaller than the silicone drop, and the beam is positioned such that its center is near the center of the silicone drop. If all of the objects analyzed are ***exactly*** the same size as the silicone drop, the results will not depend on which of the three scenarios is at play in this system. However, if objects are larger than the drop being analyzed, and either Scenario 1 or Scenario 2 is an accurate depiction of the beam/drop interaction, one would expect to observe more silicone for the larger object, purely due to an enhanced object/beam interaction volume. This appears to be what the author is suggesting when he says, “The objects analyzed had a larger spatial expansion than such a spot and, thus, sample ions may have been generated and collected from an even larger surface, which in turn caused a higher amount of sample ions per run than could be obtained from a silicone oil spot” [1, p. 517]. Similarly, if the object being analyzed is smaller than the silicone drop, one would expect to observe less silicone due to a decreased beam/sample interaction volume. Again, this appears to be what the author is suggesting when he says, “The sample spot size effect was also observed during the analysis of the SGE yellow silicone septum that was only 5 mm in diameter and was, thus, found to release only 20 μ μg of PDMS per run” [1, p. 517–518].

“Quantified” values from 15 samples are included in Table 2. Of these, signal levels that are above the highest value measured in the calibration curve are recorded for 8 out of the 15 samples. This strongly suggests that there is an interaction volume problem at hand. That is, since samples are larger than the calibrant drop size used for quantification, and since the gas stream appears to be sampling a projected area that is larger than the calibrant drop area, it is not surprising that there is much more signal observed for the objects analyzed in Table 2. Note that this problem applies not only to the eight samples that are completely off of the calibration curve, but also to all other samples that are larger in area than the silicone drops used for calibration. To rephrase, it is almost certainly the case that nearly every value of PDMS mass expressed in Table 2 is artificially high because the calibration curve shown in Figure 4 appears to have been generated from drops with a cross-sectional area that is smaller than the dimensions of the gas stream (Scenarios 1 and 2). One exception to this may be the SGE yellow silicone, which the author notes was quite small. However, since the author did not measure the dimensions of the silicone drops, there are no data to use to assess whether the SGE yellow silicone piece analyzed was larger or smaller than the drops used for calibration. A further comment on the subject of drop size: A fairly broad concentration range of silicone was applied to the glass surfaces using dichloromethane as a carrier solvent. It would be interesting to understand how both the viscosity and the surface tension of the silicone/dichloromethane solutions vary over that concentration range, and what impact this has on the resulting drop size. Silicones are extremely low surface tension materials and tend to spread on high-energy surfaces like glass. We believe it is definitely possible that the drop size varies from one concentration to the next, and, if so, this would result in variations in the sum of silicone peak intensities measured on these drops, assuming our hypothesis regarding the gas stream/drop interaction geometry is correct.

A possible nongeometric problem with the method of quantification is that it is not necessarily the case that the instrument response will be the same for a low molecular weight PDMS species versus a moderate molecular weight PDMS species (e.g., response of 8-unit oligomer versus 19-unit oligomer). Although this may be a safe assumption, a more rigorous approach would simply involve measuring the response factors of individual cyclic siloxane species to ensure that they are the same. As that experiment does not require a major resource investment, it is surprising that it was not included here. In the absence of individual oligomer standards, an alternative method of establishing response factors would have been to examine the silicone oil used as a calibration standard by gas chromatography flame ionization detection (GC-FID). After correcting the FID response for the weight percent of carbon in each oligomer (traditional FID theoretical response factor method), if the resulting corrected relative distribution of peak intensities matches that observed by DART, the assumption that the response factor of each oligomer is the same is correct.

## Problems with the Slant of the Article

The DART conditions used (300 °C or 572 °F) are significantly harsher than the recommended and normal use conditions of pacifiers, bottle nipples, and bakeware. Though the author mentions, “Admittedly, exposure of a pacifier to the DART gas at 300 °C does not exactly reflect the conditions of normal use…” [1, p. 515] and “Some difference is to be expected between the DART analysis of a silicone rubber object and its ability to release PDMS under the conditions of its normal use” [1, p. 519], the wording is very mild and not mentioned in the Abstract, Conclusions, or locations where quantitative “release” results are discussed. Moreover, the quantitative results from direct exposure of the materials to the DART-MS source is in a section labeled “Quantification of Silicone Release” [1, p. 518], which may easily be misinterpreted as representing expected human exposure under normal use conditions. Included are several examples of the strong wording used as a way to sensationalize the article:

- Abstract: “These findings indicate a potential health hazard from frequent or long-term use of such items.” “A higher level of awareness of this source of daily silicone intake is suggested.”
- Pg. 511: “When studied, the long-term exposure of humans to silicones was found to induce adverse health effects.” More through the end of this section.
- Pg. 512: “Among other organic contaminants, PDMS has been analyzed by DART-MS in agricultural biosolids [30].” – suggests PDMS is a contaminant. Additionally, none of the articles cited in the referenced review article [30] utilize DART-MS analysis.
- Pg. 512: “Thus, a substantial dose of silicones may be taken up from such articles by humans, in particular during elongated exposure under extracting conditions as in the case of pacifiers or teething rings…” His own evidence suggests that almost no silicone is released from aqueous extraction of these objects.
- Pg. 512: “The immediate strong release of silicones at the elevated temperature of DART analysis indicates a potential health risk from daily use of such silicone items.”
- Pg. 512: “The intention of this work… [is] to raise an alertness for the health implications and to initiate thinking about alternatives.”
- Pg. 515: “Baking molds are supposed to withstand heat for an elongated period… [as] confirmed by the imprinted temperature limits.” “The manufacturer’s statement is obviously intended to provide confidence for the potential user that this product is safe for use when in direct contact with food even when exposed to high temperature for about 1 hour.”
- Pg. 518: “From a consumer’s point of view, it is particularly frustrating that the baking molds and the scraper that are all designated to elongated use at high temperature belong in the group of most efficient PDMS releasers.”
- Pg. 520: “These findings indicate a potential health hazard from frequent or long-term use of such items in general.”

The substances the author detected are of a molecular weight >500 or even 1000 Da, meaning that they are of limited (if any) systemic bioavailability. A linear siloxane (dodecamethylpentasiloxane, L5; CAS no. 141-63-9; molecular weight: 384.85 g mol-1) has been already registered under [REACH (Regulation on Registration, Evaluation, Authorisation, and Restriction of Chemicals; REGULATION (EC) no 1907/2006 of the European parliament and of the council of 18 December 2006)]. The dissemination report is published at the European Chemical Agency (ECHA) [http://apps.echa.europa.eu/registered/data/dossiers/DISS-dcee80b8-2d20-1adf-e044-00144f67d031/DISS-dcee80b8-2d20-1adf-e044-00144f67d031_DISS-dcee80b8-2d20-1adf-e044-00144f67d031.html]. Since L5 has a lower molecular weight, the potential for the material to be bioavailable would be assumed to be greater than that of the higher molecular weight PDMS. The toxicological profile of L5 could then represent a “worst-case” scenario for these linear materials. No hazardous health effects have been observed in various endpoint studies, which cover acute and repeated dose toxicity, irritation, sensitization, and carcinogenic, mutagenic, reproductive toxicant (CMR) properties. Similarly, no adverse health effects have been observed with polydimethylsiloxanes (PDMS). A summary of various animal data and epidemiologic studies is given in the ECETOC JACC Report no. 55 [6] and results are discussed. No relevant toxicological effects have been observed, even following lifetime exposure of rats to PDMS by the oral route. This is also true for immune toxicological properties of PDMS. However, in the current manuscript, Gross states repeatedly that the released substances may be potential health hazards. We think that such a statement should only be made based on scientific evidence rather than raising suspicion based only on the fact that substances are emitted at an unrealistically high temperature. The “adverse effects” of silicones that the author describes in the publication (breast cancer, fibrosis, autoimmunity, inflammatory processes), are not proven or are discussed controversially. Specifically, studies on silicone breast implants have not supported a relationship to carcinogenesis. US-FDA came to the conclusion that “There is no apparent association between silicone gel-filled breast implants and connective tissue disease, breast cancer, or reproductive problems. Associations that are very rare or that take many years to manifest may not be detected using currently available data” [7; see also 6]. It must be noted that Reference 1 in the paper by Gross does not discuss any correlation between silicones and breast cancer, so the author’s statement that “health effects of silicones … are discussed in the context of breast cancer” [8] with reference to [1] is wrong.

Concerning the exposure situation in the Gross publication, the relevant physiological route of exposure (oral) should be given priority. No toxicity up to the highest applied dose after oral exposure has been observed in animal tests with L5 and PDMS.

## DART-MS Conditions are Vastly Different than Real-World Use Conditions and the Data Show It

The author did perform extraction experiments to mimic in-use conditions of a bottle nipple, a pacifier, and baking molds. The pacifier and nipple extractions resulted in nondetectable PDMS levels when analyzed by DART-MS. This is in stark contrast to the 300 °C DART-MS analysis of the materials, which gave rise to 35 to >100 μg PDMS. The pacifier and nipple extract results are not only excluded from the summary in Table 2, but the direct comparison is never discussed in the article. Furthermore, since PDMS was not observed from the normal use extracts by DART-MS, the picture of the pacifier with the overlay of the mass spectrum of a high molecular weight PDMS species used in both the article and as the journal cover image is certainly misleading and provocative. An additional criticism of this image is that it shows the structure of a trimethyl-endcapped linear dimethylsiloxane, while the included mass spectrum is of a homologous series of cyclic dimethylsiloxanes. Though this is a somewhat minor point, it is yet another example of the poor quality of this article. An additional example of the overstatement of PDMS exposure expressed in this article comes from the analysis of baking molds. Though we do not know the exact interaction volume of the gas stream with the baking molds, it is clear that the extraction experiments used a substantially larger quantity of material (1 g bakeware in 2 mL oil) than was sampled in the DART experiments. The observation of a 30–40× increase in calculated PDMS mass in the DART-MS experiments versus the extraction experiments clearly demonstrates that extraction by DART-MS does not represent real-world conditions.

## Comments on Surface Degradation

On p. 515, the author states, “None of the items did show visible marks, discoloration, or the like after analysis. Overall, the conditions chosen for analysis seem not to affect the surface integrity of the objects” [1]. At this time, we have no direct evidence to confirm or dispute the claim that the analysis conditions did not cause surface degradation. However, the author’s chosen method of determining whether degradation occurred is grossly inadequate. Visual inspection of a surface briefly exposed to the DART source is unlikely to show visible damage, since even if damage is present, it is likely confined to a very shallow surface depth. However, chemical analysis using X-ray photoelectron spectroscopy or topographic analysis using either atomic force microscopy or white-light interferometry would directly show whether any chemical or topographic changes were induced upon DART gas exposure, and these would be much better methods to assess degradation than simply looking at the samples.

Karluss Thomas

Executive Director

Global Silicones Council

## References

[1] Gross JH (2015). Analysis of silicones released from household items and baby articles by direct analysis in real time-mass spectrometry. J. Am. Soc. Mass Spectrom..

[2] Zhao Y, Lam M, Wu D, Mak R (2008). Quantification of small molecules in plasma with direct analysis in real time tandem mass spectrometry, without sample preparation and liquid chromatographic separation. Rapid Commun. Mass Spectrom..

[3] Yu S, Crawford E, Tice J, Musselman B, Wu JT (2009). Bioanalysis without sample cleanup or chromatography: the evaluation and initial implementation of direct analysis in real time ionization mass spectrometry for the quantification of drugs in biological matrixes. Anal. Chem..

[4] Nilles JM, Connell TR, Durst HD (2009). Quantitation of chemical warfare agents using the direct analysis in real time (DART) technique. Anal. Chem..

[5] Saang'onyo D, Selby G, Smith DL (2012). Validation of a direct analysis in real time mass spectrometry (DART-MS)method for the quantitation of six carbon sugars in a saccharification matrix. Anal. Methods.

[6] ECETOC (European Centre for Ecotoxicology and Toxicology of Chemicals) JACC (Joint Assessment of Commodity Chemicals) Report No. 55, Linear PDMS CAS No. 63148-62-9. 2nd edition; Brussels, December (2011)

[7] FDA Update on the Safety of Silicone Gel-Filled Breast Implants. Center for Devices and Radiological Health U.S. Food and Drug Administration, June (2011)

[8] Reyal F, Feron JG, Leman Detour S, Pourcelot AG, Valentin M, Phillippe AC, Levy-Zauberman Y, Agman A, Monier S, Blondel A, Cothier-Savey I, Guihard T, LeMasurier P, Fitoussi A, Couturaud B (2013). The impact of poly implant prothese fraud on breast cancer patients: a report by the Institut Curie. Plast. Reconstr. Surg..

